# News and Notes

**Published:** 1915-05

**Authors:** 


### NEWS AND NOTES

### CANCER EXPERTS TO SPEAK IN VERMONT.

#### Program of Meetings at Rutland, St. Johnsbury and Montpelier.

Speakers of national prominence have been obtained by the Vermont State Medical Society for its proposed series of educational meetings on cancer to be held in the principal cities of that state early next month. The program calls for an identical series of morning, afternoon and evening meetings to be held at Rutland on June 8th, Burlington on June 9th, St. Johnsbury on June 10th and Montpelier on June 11th. In the morning of each day a clinic will be held by the visiting physicians to which local doctors will bring patients for consultation. In the afternoon, the meetings will be for the medical profession, but the evening meetings will be open to the public and the addresses will be popular in character.

Dr. Francis Carter Wood, of New York, will speak at Burlington on the afternoon and also on the evening of June 9th and will take part in the clinic at Burlington on June 9th and at St. Johnsbury on June 10th. Dr. Wood is the

Director of the Cancer Research work of Columbia University, which enjoys, in the George Crocker Special Research Fund, the most considerable endowment for cancer research in this country, if not in the world. In the special laboratory buildings recently opened on Morningside Heights the search into the mysteries of cancer is being pursued with all the equipment and methods known to science. One of the features of the work at the Columbia Laboratory is the facilities offered to practicing physicians to keep themselves informed as to the latest developments in the methods of diagnosis of cancer in its early stages.

Dr. J. M. Wainwright, of Scranton, Pa., will take part in the morning clinics at Burlington on June 9th, and at St. Johnsbury on June 10th and will address the physicians at the afternoon meetings in these cities and also in Rutland on June 8th and Montpelier on June 11th. Dr. Wainwright has long been the Chairman of the Cancer Commission of the Pennsylvania State Medical Society, under whose auspices one of the first educational movements in America in regard to this disease was organized and carried on. Both Dr. Wainwright and Dr. Wood are directors of the American Society for the Control of Cancer which is co-operating with the Vermont Medical Society in the arrangements for these meetings.

Dr. Charles F. Dalton, Secretary of the Vermont State Board of Health, will speak at the public evening meetings on all four days, officially representing the State Health Department.

Dr. W. S. Bainbridge, of New York, will be the principal speaker at the evening meetings at Rutland, St. Johnsbury and Montpelier.

---

### MEETING OF THE CHATTAHOOCHEE MEDICAL ASSOCIATION.

A splendid program has been arranged for the next meeting of the Chattahoochee Valley Medical Association which is to be held in Opelika, Alabama, and it is earnestly desired that a full attendance be present. No matter whether you are a member or not, you will receive a cordial welcome and see some old-fashion Southern hospitality. There will be a number of interesting papers with free discussion.

The social features will also be well worth while. Dr. Hansell Crenshaw is chairman of the program committee and has arranged the necessary details for a very successful meeting.

---

### ATLANTA ROENTGEN RAY SOCIETY.

Regular Meeting May 3rd, 1915.

By R. R. Daly, M. D.

Dr. S. L. Silverman made a Preliminary Report of Steoscopic Studies in Dental Radiology.

It covers some work he has been doing and observing in New Orleans and other cities and paves the way to exact demonstrations as soon as apparatus is perfected and the technique simplified. Kells and Matass in New Orleans are doing fine work in this direction and already their stereoscopic pictures make the jaw look like a "glass case" so far as deciding upon the relations of the teeth are concerned. The stereoscopic picture is necessary when teeth are impacted or so irregular that removal is demanded. Without it one can not well decide which tooth should be taken away. The depth of view secured by the apparatus at hand is remarkable.

Dr. E. D. Highsmith spoke of his Observations of the Use of the X-Ray in Europe. These will appear elsewhere in The Journal.

Dr. G. M. Niles said that when it comes to progress in X-Ray work, we have to look to the dentists for a great deal of it. It serves to unite the two branches of the healing profession and to make the relations more profitable to all.

The use of the screen in examination of the thorax and the observation of the heart and lungs is interesting and presents new problems. For instance, the scars of old and healed tubercular lesions in the lung tissue may exactly resemble the shadows of the new lesions and the operator must learn someway to make the distinction. In the study of large malformations or malpositions of the heart or the aorta, the physical diagnostician has to use much time and patience before he is secure in his conclusions and even then another observer may differ with him in some degree. With the X-Ray all this can be determined beyond a peradventure of a doubt in a very brief examination of or a few seconds' exposure to a plate. The technique of X-Ray work in the body and deeper structures is improving and growing rapidly. The soft rays cause burns and exposure of four minutes to these is dangerous. Brevity of examination is demanded on every count. One sees too much and misleads himself in a prolonged study of a screen. He showed plates portraying gall stones which were ultimately found at the operation. In one plate one stone was shown and in the other, twelve stones were indicated. These were correct. He also showed plates of kidney stones and said that he thought from their density that they were oxalite of lime. In answer to a question he said that the relative density of the various stones found in the kidney were as follows: Oxalate of Lime, Cystine, Phosphates, Mixed Phosphates, Mixed Urates, and finally what are almost impossible to see—Pure Urates.

Highsmith said that he thought we should make screen work a routine process and do so much of it that we could be thoroughly familiar with it and be able to determine everything in the pictures quickly and accurately. In plate work, the fixing of the patients with shot and sand bags is a European procedure that we might copy. The personal equation is important in interpreting plates. The man who made them knows better than anyone else all the points that entered into their construction and he can decide whatever is advantageous. The Germans use many coil machines as well as transformers and say they like the American makes better than any others.

